# Treatment Patterns in Patients with Metastatic Melanoma: A Retrospective Analysis

**DOI:** 10.1155/2014/371326

**Published:** 2014-05-05

**Authors:** Zhongyun Zhao, Song Wang, Beth L. Barber

**Affiliations:** Amgen Inc., Thousand Oaks, CA 91320, USA

## Abstract

*Objective.* To describe treatment patterns and factors influencing treatment in a real-world setting of US patients with metastatic melanoma (MM). *Methods.* This was a retrospective claims-based study among patients with MM diagnosed between 2005 and 2010 identified from MarketScan databases. *Results.* Of 2546 MM patients, 66.8% received surgery, 44.7% received radiation, 38.7% received systemic therapies, and 17.7% received all modalities. Patients with lung, brain, liver, or bone metastases were less likely to undergo surgery (all *P* < 0.0001); patients with lung (*P* = 0.04), brain (*P* < 0.001), or liver metastases (*P* = 0.03) were more likely to receive systemic therapies; patients with brain (*P* < 0.0001) or bone metastases (*P* < 0.0001) were more likely to receive radiation therapy. Oncologists were more likely to recommend systemic therapy (*P* < 0.0001) or radiation (*P* < 0.0001), while dermatologists were more likely to recommend surgery (*P* = 0.002). Monotherapy was the dominant systemic therapy (82.4% patients as first-line). *Conclusions.* Only 39% of MM patients received systemic therapies, perhaps reflecting efficacy and safety limitations of conventional systemic therapies for MM. Among those receiving systemic therapy, monotherapy was the most common approach. Sites of metastases and physician speciality influenced treatment patterns. This study serves as a baseline against which future treatment pattern studies, following approval of new agents, can be compared.

## 1. Introduction

Melanoma has the fastest rising incidence rate compared with that of any other malignancy [1]. Metastatic melanoma (MM) is the most aggressive form of skin cancer and generally has a poor prognosis with a median overall survival of 6–10 months and a 5-year survival of 5–10%, depending on the location of the metastasis [2], compared with 5-year survival rates of 98.2% and 62.4% for localised and regional melanoma, respectively [3].

Prior to the approval of ipilimumab and vemurafenib in 2011, only two therapies were approved by the US Food and Drug Administration for treatment of MM, dacarbazine and high-dose interleukin-2 (HD IL-2). Since its approval in the 1970s, dacarbazine monotherapy has been commonly used for the treatment of MM [4], although its use is limited by a low response rate [5, 6] as well as a lack of an overall survival benefit and significant toxicity [7]. HD IL-2 is associated with durable responses in only a small percentage of carefully selected patients [8] and is limited by severe toxicities [9].

Prior to the introduction of the newer agents, treatment guidelines recommended resection, observation, or systemic therapy for patients with resectable stage IV melanoma. For those with unresectable stage IV disease, palliative resection and/or radiation for symptomatic patients or best supportive care can be considered; some of these patients can be treated with systemic therapy or enrolled into a clinical trial [10–12]. This lack of preferred conventional treatments most likely reflects the lack of efficacy of conventional MM therapies [10, 11], and is likely to change as experience with newer agents increases and additional agents become available.

It is therefore increasingly important to understand the factors that influence treatment decisions in patients with MM. No study has explored administrative data from a real-world clinical setting to characterise treatment patterns in this population. To identify the factors that influence treatment choices, we examined retrospective claims data prior to the introduction of the newer agents from the commercially insured population to document real-world treatment patterns in patients with MM in the USA. This study provides a baseline for comparison to future analyses as new agents become available.

## 2. Patients and Methods

### 2.1. Data Source

This was a retrospective claims-based study of patients with MM using administrative claims (medical and pharmacy) taken from the Thomson Reuters MarketScan Commercial Claims and Encounter database and the Medicare Supplemental and Coordination of Benefits database, between 1 January 2005 and 30 June 2010. These data included the full continuum of care under a variety of plan types across all settings (physician office visits, emergency room visits, inpatient hospital stays, outpatient visits, and outpatient pharmacy claims). The study population had a similar overall age distribution to that of a nationally representative population in Medical Expenditure Panel Survey.

### 2.2. Study Population

Patients who were eligible for inclusion in the study were those with at least two outpatient (or at least one inpatient) melanoma diagnoses (ICD-9-CM: 172.xx, V10.82) and at least two outpatient (or at least one inpatient) diagnoses for metastases (ICD-9-CM: 197.xx, 198.xx). Thus, patients who were included in this study would have distant metastases. If patients were identified based on outpatient diagnoses, the two outpatient diagnoses for melanoma and the two diagnoses for metastases were required to be at least 30 days apart. The first metastasis date should be no more than 30 days prior to, or any time after, the first date of melanoma diagnosis, and the index date was the first date of diagnosis of metastases. Patients who had other primary malignant tumours prior to diagnosis of melanoma, those who were younger than 18 years old at the index, and those with a preindex period of <6 months were excluded from the analysis.

### 2.3. Study Measures

The main study measures included identification of the percentage of patients who received cancer-related surgery, radiation, or systemic therapy for the treatment of MM. The type of surgery includes incision, excision, repair, shaving, removal, and destruction, etc., and the sites of surgery include skin, soft tissue, lymph nodes, lung, liver, and brain. Systemic drug treatments were identified using the Healthcare Common Procedure Coding System, the International Classification of Diseases (9th Revision), Clinical Modification (ICD-9-CM) procedure codes, or National Drug Code for any of the drug agents in postindex and included Bacillus Calmette-Guérin, carboplatin, carmustine, cisplatin, dacarbazine, docetaxel, granulocyte-macrophage colony-stimulating factor (GM-CSF), IL-2, interferon alfa-2b, paclitaxel, temozolomide, and vinblastine.

Further analysis of patients receiving systemic therapy was performed to identify the type of systemic drugs used by lines of therapy and the duration of therapy. Here, a treatment regimen was defined as one or more agents administered within a 4-day period, with the condition that all elements were administered more than once within 28 days. In the event that a new drug was added to a regimen within 28 days of the start of a line of therapy, this was considered an addition to the existing treatment regimen rather than a new line of therapy. The end of a given line of therapy was defined as either a 90-day gap in treatment or initiation of a new regimen that was not merely the addition of a new drug to the existing regimen.

Other study measures included demographic factors (such as age, gender, and region), type of health insurance plan, baseline Charlson comorbidities, metastatic site at index date, and speciality of treating physician within 90 days after index date. Each patient was followed from index date to death, termination of health insurance, or until end of database availability (30 June 2010), whichever occurred first.

### 2.4. Statistical Analysis

Factors influencing use of surgery, systemic therapy, or radiation were assessed by logistic regressions. Mean and standard deviation (SD) were calculated for continuous variables, while percentages were calculated for categorical variables for patient demographic and clinical characteristics. Treatment patterns for systemic therapies including percentage of patients receiving each drug of interest and duration of therapy were presented descriptively by graphs. All data were analysed using SAS programs (SAS Institute Inc., Cary, NC, USA) organised under UNIX using SAS version 9.2.

## 3. Results

### 3.1. Patient Demographics

A total of 2546 patients with MM were identified in the MarketScan databases with a mean age at index of 60.6 (±14.0) years, 6.8% of whom were 18–<40 years old, 58.5% were 40–<65 years old, and 34.7% were 65 years and older. The main sites of metastases were lung (21.2%), brain (18.7%), distant areas of skin (11.4%), bone (11.2%), liver (10.0%), and other sites (27.5%). Other sites include gastrointestinal tract, adrenal gland, genital, kidney, and ovary. The mean length of follow-up (SD) was 322.4 days (356.3) (Table 1).

### 3.2. Overall Patterns of Care

Overall, 66.8% of patients received cancer-related surgery, 44.7% received radiation, 38.7% received systemic drug therapies, and 17.7% of patients received all three treatments.

As shown in Table 2, a number of factors appear to influence treatment decisions in patients with MM. These include site of metastases, speciality of treating physician, and type of health insurance. Specifically, patients with lung, brain, liver, or bone metastases were significantly less likely to undergo surgery (all *P* < 0.0001), whereas patients with lung, brain, or liver metastases were more likely to receive systemic therapy (*P* = 0.04, *P* < 0.001, and *P* = 0.03, resp.), and patients with brain or bone metastases were significantly more likely to receive radiation therapy (*P* < 0.0001). In addition, patients who were treated by oncologists were significantly more likely to receive systemic therapy (*P* < 0.0001) or radiation (*P* < 0.0001), whilst those treated by dermatologists were more likely to receive surgery (*P* = 0.002). Finally, patients with comprehensive medical insurance were more likely to receive radiation (*P* = 0.031) than those with insurance provided by a Health Maintenance Organization (HMO), the Preferred Provider Organization (PPO), or those with a Point-of-Service Plan (POS).

### 3.3. Treatment Patterns in Patients Receiving Systemic Drug Therapy

**Figure 1:**
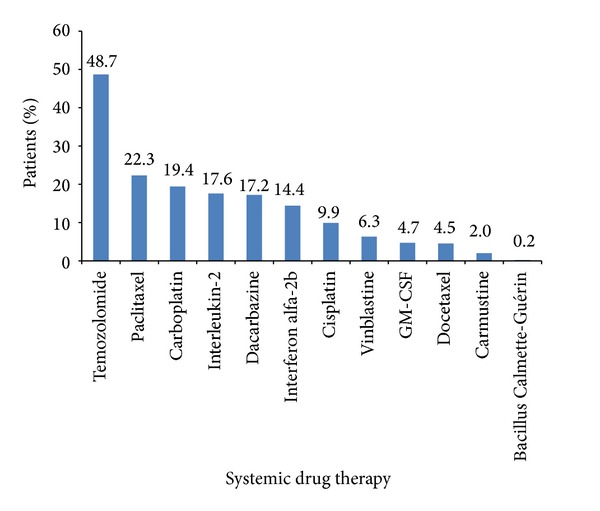
Treatment patterns among patients who received systemic drug treatment.

The treatment patterns in patients receiving systemic drug therapy across all lines of therapy are summarised in Figure 1. Among the 985 patients who received systemic drug therapy, the most prevalent agent was temozolomide (48.7%), followed by paclitaxel (22.3%), carboplatin (19.4%), IL-2 (17.6%), and dacarbazine (17.2%). The site of metastases influenced the type of systemic regimen received; for example, patients with brain metastasis were more likely to receive temozolomide and did not receive treatment with IL-2.

**Figure 2:**
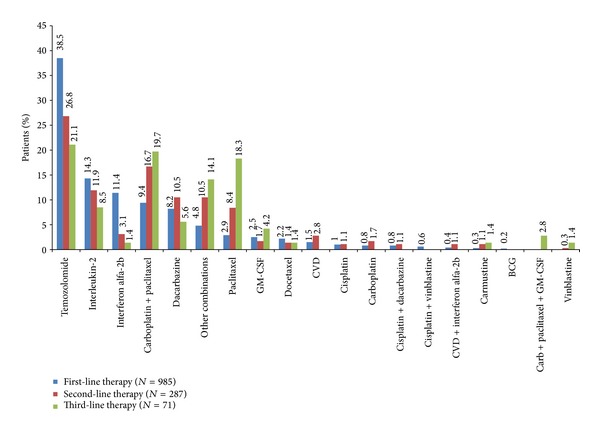
Treatment patterns by line of therapy.

Further examination of the treatment patterns by lines of systemic drug demonstrated 82.4% of patients received monotherapy as the first documented therapy after diagnosis, with temozolomide (38.5%) and dacarbazine (8.2%) being the most commonly used chemotherapy agents. IL-2 (14.3%) and interferon *α*-2b (11.4%) were the predominant immunotherapies, and carboplatin + paclitaxel was the most common combination therapy (9.4%) (Figure 2).

**Figure 3:**
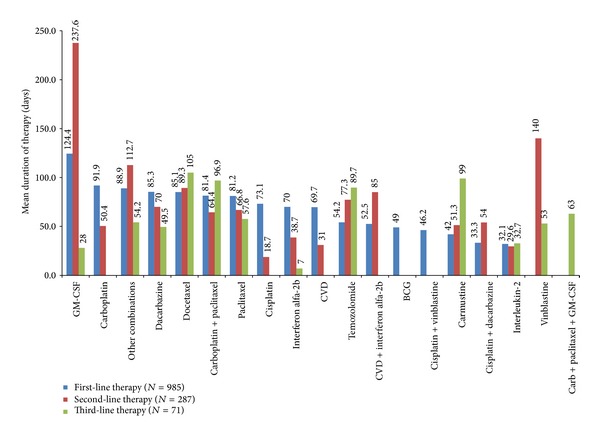
Treatment duration by line of therapy.

The mean duration of treatment in the first-line setting was 63 days, ranging from 32 days on IL-2 to 124 days on GM-CSF (Figure 3). Of the 985 patients who received first-line therapy, 287 (29.1%) patients subsequently received second-line treatment. In this setting, temozolomide (26.8%) was again the most commonly used agent, followed by carboplatin + paclitaxel (16.7%), IL-2 (11.9%), dacarbazine (10.5%), and paclitaxel (8.4%), with the majority of patients receiving monotherapy (68.0%) (Figure 2). The mean duration of treatment was 70.7 days, ranging from 19 days on cisplatin to 238 days on GM-CSF (Figure 3).

Of the 287 patients who received second-line treatment, 71 (24.7%) went on to receive third-line therapy. In this setting, monotherapy was also predominant (63.4%), with patients receiving single-agent chemotherapy with temozolomide (21.1%), paclitaxel (18.3%), or dacarbazine (5.6%) (Figure 2). Combination of carboplatin + paclitaxel was used in 19.7% of patients and single-agent immunotherapy with IL-2 was used in 8.5% of patients. The mean duration of treatment was 68.5 days, ranging from 7 days on interferon *α*-2b to 105 days on docetaxel (Figure 3).

## 4. Discussion

Analysis of administrative claims data allows examination of treatment patterns in the real-world setting, reflecting the diversity of the agents used in clinical practice. This study therefore aimed to document real-world treatment patterns in patients with MM, with a focus on the types of systemic drug therapies used in first-, second-, and third-line therapy. The large study population of 2546 patients provided a robust data set with which to examine these aims.

Examination of MarketScan databases revealed that the majority of patients with MM received cancer-related surgery (66.8%). Only a relatively low proportion of patients (38.7%) received systemic drug therapy, perhaps reflecting the lack of efficacy with conventional treatment agents. The current study also identifies a number of factors, including sites of metastases, speciality of the treating physician, and the type of insurance held, which appear to influence whether a patient receives surgery, systemic drug therapy, or radiation.

As expected, given the lack of consensus on the effective use of conventional systemic drugs in MM, examination of the patterns of systemic drug therapy revealed that a variety of chemotherapy agents were used, with off-label temozolomide monotherapy being the most prevalent agent among patients who received drug treatment (48.7%). The increased use of temozolomide over dacarbazine (17.2%) observed in this study is likely to be a result of the greater ease of administration of temozolomide due to its oral availability and its ability to cross the blood-brain barrier [13], with neither showing overall survival benefit. As expected, other commonly used treatments consisted of immunotherapies, such as IL-2 and interferon alfa-2b, and single-agent chemotherapy with paclitaxel or carboplatin. Given that conventional chemotherapy agents are associated with poor response rates [5, 6] or, in the case of IL-2, significant toxicity [9, 14], these findings represent an unmet need for more effective treatment alternatives in patients with MM.

When the types of agents used were categorised according to lines of treatment, off-label temozolomide monotherapy remained the most common approach across all three lines of treatment (first-, second-, and third-line). It is interesting to note that monotherapy in general was prevalent across all three lines of therapy, reflecting that although combination chemotherapy and biochemotherapy have been shown to improve objective response rates in some cases, they tend not to extend survival and are associated with greater toxicity [4, 7, 15]. While GM-CSF was not the most frequently used treatment option, it was associated with the longest duration of treatment in first- and second-line. The long treatment duration with GM-CSF might be because it has a tolerable safety profile and patients who received GM-CSF likely were young and had low baseline comorbid disease burden [16].

It is important to acknowledge some limitations of the study, including its reliance on administrative claims submitted solely for the purposes of medical reimbursement. The databases for the commercially insured patient population are not necessarily representative of all patients with MM in the USA. The analysis of specific systemic agents used is also limited by the varying detail with which systemic therapies are coded for purposes of medical care reimbursement. The impact of potential misclassification bias stemming from analyses of claims data has been described previously [17, 18]. In addition, the number of patients identified as receiving systemic drug therapy may be underrepresented since information was not recorded for patients enrolled in clinical trials. Also, in the analysis of subsequent treatments observed, patients with a short follow-up period might not have had their second or third line of therapy captured and so the percentage of second- and third-line therapy might be underestimated. Finally, the relevance of this study will depend on the success of agents that have recently come to market, including ipilimumab and vemurafenib. These are likely to pave the way for the next generation of targeted therapies and therefore may change the way MM is treated in the future. As such, it would be interesting to assess how real-world treatment patterns in MM change over the coming years.

## 5. Conclusions

This study described the treatment patterns in patients with MM in the USA, showing the variety of systemic agents that were used during the study period. These findings demonstrate the need for more efficacious and tolerable treatment alternatives for MM. Furthermore, this study provides an overview of how patients with MM were managed before the newer agents were approved and serves as a baseline for future studies of this type to assess how newer agents are adopted into clinical practice in real-world settings and how they influence the treatment patterns of patients with MM.

## Figures and Tables

**Table 1:** Patient demographic and clinical characteristics.

	Total (*n* = 2546)
Age, mean (SD)	60.6 (14.0)
18–<40, %	6.8
40–<65, %	58.5
65 and older, %	34.7
Female, %	36.5
Health insurance plan, %	
Comprehensive	20.9
HMO	12.3
POS	6.7
PPO	56.1
Other plans	4.0
Medicare beneficiaries, %	33.0
Regions, %	
North east	10.5
North central	29.1
South	41.6
West	18.8
Metastasis site at index, %	
Lung	21.2
Brain	18.7
Distant part of skin	11.4
Bone	11.2
Liver	10.0
Other sites	27.5
Charlson comorbidity index (excluding cancer), mean (SD)	0.82 (1.17)
Specialty of treating physician within 90 days after index, %	
Oncologist	52.1
Dermatologist	15.0
Length of follow-up, mean days (SD)	322.4 (356.3)

**Table 2:** Logistic regression of factors influencing systemic therapy, surgery, and radiation.

	Systemic therapy			Surgery			Radiation		
	Odds ratio	*P* value	95% confidence interval	Odds ratio	*P* value	95% confidence interval	Odds ratio	*P* value	95% confidence interval
Age at index	0.970	<0.0001	[0.961, 0.980]	0.986	0.013	[0.976, 0.997]	0.984	0.001	[0.974, 0.994]
Female	0.787	0.008	[0.658, 0.939]	0.887	0.216	[0.734, 1.073]	0.727	0.001	[0.607, 0.870]
Type of insurance plan									
Comprehensive	1.168	0.529	[0.720, 1.897]	1.160	0.563	[0.701, 1.918]	1.721	0.031	[1.050, 2.281]
HMO	1.105	0.688	[0.679, 1.799]	0.676	0.134	[0.404, 1.129]	1.190	0.500	[0.718, 1.973]
POS	0.805	0.423	[0.474, 1.368]	0.981	0.946	[0.558, 1.725]	1.543	0.120	[0.894, 2.663]
PPO	1.041	0.858	[0.673, 1.610]	1.053	0.827	[0.665, 1.667]	1.489	0.084	[0.948, 2.338]
Medicare beneficiary	0.755	0.071	[0.557, 1.025]	0.902	0.537	[0.651, 1.250]	0.850	0.301	[0.624, 1.157]
Region									
Northeast	0.898	0.528	[0.643, 1.254]	1.541	0.020	[1.069, 2.222]	1.044	0.806	[0.742, 1.469]
North central	1.026	0.843	[0.793, 1.328]	1.040	0.783	[0.786, 1.377]	1.019	0.889	[0.782, 1.328]
South	0.857	0.214	[0.672, 1.093]	1.044	0.748	[0.803, 1.356]	1.070	0.594	[0.835, 1.371]
Metastasis site at index									
Lung	1.292	0.039	[1.013, 1.647]	0.418	<0.0001	[0.319, 0.548]	0.920	0.498	[0.723, 1.171]
Brain	1.598	0.000	[1.239, 2.062]	0.498	<0.0001	[0.377, 0.658]	6.388	<0.0001	[4.851, 8.412]
Distant part of skin	1.054	0.730	[0.782, 1.420]	0.867	0.451	[0.598, 1.257]	0.929	0.628	[0.691, 1.250]
Bone	0.830	0.245	[0.606, 1.136]	0.302	<0.0001	[0.219, 0.417]	2.628	<0.0001	[1.966, 3.512]
Liver	1.426	0.026	[1.044, 1.946]	0.280	<0.0001	[0.201, 0.391]	0.624	0.005	[0.451, 0.865]
Charlson comorbidity index (removed cancer)	0.985	0.683	[0.918, 1.058]	1.155	0.000	[1.068, 1.250]	1.065	0.084	[0.992, 1.143]
Specialty of treating physician within 90 days after index									
Oncologist	1.534	<0.0001	[1.288, 1.828]	1.051	0.606	[0.870, 1.269]	1.581	<0.0001	[1.324, 1.887]
Dermatologist	0.902	0.406	[0.708, 1.150]	1.586	0.002	[1.181, 2.129]	0.808	0.089	[0.632, 1.033]
Length of follow-up	1.001	<0.0001	[1.001, 1.001]	1.003	<0.0001	[1.002, 1.003]	1.000	0.163	[1.000, 1.000]
